# Biodegradable and self-expanding metal stents in the treatment of children with strictures after esophageal atresia repair

**DOI:** 10.1007/s10388-026-01184-5

**Published:** 2026-02-24

**Authors:** Johannes Melchior, Frauke Blaum, Oliver J. Muensterer

**Affiliations:** 1 Department of Pediatric Surgery, Dr. von Hauner Children’s Hospital, Medical Center of the Ludwig Maximilian University Munich, Lindwurmstrasse 4, 80337 Munich, Germany; 2 Department of Pediatric Surgery and Pediatric Urology, Braunschweig Hospital, Salzdahlumer Strasse 90, 38126 Brunswick, Germany; 3 Department of Pediatric Surgery, Medical Center of the Johannes Gutenberg University, Langenbeckstrasse 1, 55131 Mainz, Germany

**Keywords:** Esophageal atresia, Caustic ingestion, Esophageal stenosis, Esophageal stenting, Biodegradable stents, Self-expanding metal stents

## Abstract

**Purpose:**

This study compared the efficacy and complications of biodegradable stents (BDS) versus self-expandable fully covered metal stents (SEMS) in pediatric patients with strictures after esophageal atresia repair.

**Methods:**

The charts of children with esophageal atresia (EA) undergoing stent treatment for anastomotic stricture were retrospectively reviewed. Primary outcomes included time to reintervention (TTR) and procedural success, defined as no necessity of further intervention with a patent esophageal lumen at the time of follow-up. Secondary outcomes included stent-related complications.

**Results:**

From November 2016 to March 2024, a total of 22 BDS and 26 SEMS were placed in 15 patients for strictures. Median TTR was 77 days for BDS vs. 49 days for SEMS. BDS-treated patients had a 64% lower relapse risk (HR: 0.36, CI: 0.19–0.7, *p* < 0.003). At the end of individual follow-up (median: 6 months), exclusive stenting succeeded in 4 patients, stenting with other minimally invasive procedures in 2, and with surgery in 2. Migration (18.8%) was more less frequent with BDS compared to SEMS (OR: 0.11, CI: 0.00–0.97, *p* < 0.028), while there was a trend towards more granulation tissue formation with BDS (OR: 3.48, CI: 0.99–24.2, *p* = 0.052).

**Conclusion:**

Although relapse occurs frequently in the long term, stenting may offer an alternative to assure esophageal patency in the medium term with only few and minor associated complications, notably stent migration and granulation tissue potentially causing restenosis. If placed for recalcitrant stricture, longer periods free of interventions are achieved in comparison to iterative dilatation, particularly when using BDS.

**Table:** 

**What is Known**
Recalcitrant strictures, leaks and fistula formation are challenges associated with esophageal atresia repair and other conditions.
There is anecdotal evidence that stents may represent a treatment alternative.
**What is New**
Stents are useful adjuncts for benign esophageal conditions in children.
Post-interventional patency at 90 days is achieved in up to one third of patients.
Stents afford longer intervention-free intervals, and decrease hospitalization times.
Self-expanding metal stents are more likely to dislocate, while biodegradable stents are associated with granulation formation.
These factors should be taken into account when choosing the optimal stent for a particular patient.

## Introduction

Diseases of the esophagus in infants and children are often complex and associated with prolonged morbidity. When surgical intervention is required, postoperative stricture, anastomotic leak due to suture dehiscence, and tracheoesophageal fistula are among the most common complications [1]. Strictures are most common after caustic injuries and esophageal atresia repair [2]. Management is highly individualized, including balloon dilations, topical agents, and endoluminal stents [3]. Stents are available in various types, differing in material (resorbable vs. non-resorbable), structure (covered vs. non-covered), length, and diameter [4].

Stents are a well-established treatment in adults for both malignant and benign esophageal diseases [5]. However, success rates vary significantly from anywhere between zero and eighty percent [6]. A meta-analysis of 18 studies in adults reported a pooled success rate of 40.6% across all stent types, with no significant differences in success or complications [7]. In pediatric patients, stents were first used in 1976 to prevent strictures following caustic injuries [8]. A systematic review including 97 children and 160 stents found no further treatment was needed for 52% of strictures. The authors concluded that stents may offer long-term success but often serve as a bridge to surgery for complex lesions [9]. Pediatric stent use has recently increased due to advancements in materials, including covered non-resorbable self-expanding metal and plastic stents (SEMS and SEPS) and biodegradable stents (BDS) [10]. BDS are made of polydioxanone and have been less studied in children. They dissolve over about three months, allowing for a longer dwell time and eliminating the need for endoscopic removal [11]. While adult data suggest benefits, their efficacy in children has remained unclear.

The use of stents in pediatric esophageal atresia surgery is controversial, largely due to a lack of sufficiently conclusive studies with larger case numbers. Additionally, dedicated pediatric stents are rare, so adult-sized stents—often originally designed for other indications—are used off-label in pediatric patients. This can affect procedural success and increase the risk of complications like migration due to size mismatch [12, 13]. The highly variable approaches and differing experience levels among treating centers also play a crucial role.

This study retrospectively analyzed all esophageal stents placed in pediatric patients at our institutions. The primary aim was to evaluate and compare the clinical performance of BDS and SEMS, addressing the lack of robust data on their use, efficacy, and complications. The secondary aim was to formulate clearer recommendations for stent use in pediatric patients, particularly regarding the choice between BDS and SEMS.

## Methods

All children with esophageal atresia (EA) who underwent stent treatment at two academic medical centers from November 2016 to March 2024 were retrospectively included. Inclusion criteria were age below 18 years and complete datasets with at least 3 months of follow-up. Patients were excluded if the stent was placed externally.

All stent interventions were performed under general anesthesia and endoscopic visualization. For stent placement, a guidewire was advanced, and the stent was placed under fluoroscopic guidance to ensure it fully covered the lesion. A final endoscopy was performed to confirm patency.

The choice of stent type was based on indication, size, availability, and shared decision making between surgeon and family. In patients that lived in another country or at a greater distance from the hospital, biodegradable stents were preferred to avoid another visit for stent removal and thereby decrease the amount of travel. The analysis focused on strictures after esophageal atresia repair. Leaks, fistulas, and combined indications were excluded. Isolated strictures were treated with both BDS and SEMS. For patients from remote areas, BDS were preferred due to their dissolution. All resorbable stents were uncoated, while all non-resorbable stents were coated. Due to limited availability, adult-sized biliary and tracheal stents were used off-label in the non-resorbable group. The resorbable stents were SX-ELLA-degradable BD Stents, made of polydioxanone that degrades within 90–120 days. The non-resorbable stents included silicone-covered biliary stents (BONASTENT®) and other types.

Data from electronic medical records included sex, age, weight, primary diagnosis (EA or caustic injury), comorbidities, and prior/subsequent surgical interventions. For each stent, procedure and removal dates were recorded, along with indications for placement and removal. Stent dwell time was calculated. For patients with multiple stents, the interval between placements and total number of stents were analyzed. Stents were categorized by type and size. Reinterventions were documented by indication and type.

Time to Reintervention (TTR) was the interval between stent placement and the first reintervention due to clinical relapse. A single patient could contribute multiple events. Stent removal was not a reintervention unless it was required due to a clinical relapse. Stent-related complications were categorized as migration, occlusion, erosion, granulation tissue, bleeding, new fistula, or exacerbated reflux.

Therapeutic success was defined as the absence of any further intervention combined with a patent esophageal lumen, confirmed by endoscopy, contrast study, or clinical assessment. Short-term success was assessed at 30, 60, and 90 days, while long-term success was determined at the last follow-up.

This retrospective study relied on existing medical records, so blinding was not feasible. Data were extracted by trained research personnel using standardized forms to minimize bias. All entries were independently reviewed. All procedures at both centers were performed by the same lead surgeon and an experienced team. Outcome evaluation was based on objective, predefined clinical parameters.

Continuous variables are presented as mean with SD or as median with IQR. Categorical variables are given as absolute (n) and relative (%) frequencies. A *p*-value < 0.05 was considered statistically significant. A Cox regression, adjusted for intra-patient correlation, analyzed the impact of stent type and indication on recurrence and TTR. The proportional hazards assumption was verified. Associations between stent types and complications were analyzed using the Fisher’s exact test. Odds ratios (OR) derived from Fisher’s exact test with confidence intervals were calculated.

The study was approved by the local ethics board (registration number 22–0248). Written informed consent for the procedure was obtained from all families.

## Results

In the two participating medical centers, a total of 22 patients underwent 82 stent placements during the study interval. After exclusions of a patient with stent placement for caustic injestion, 2 patients who had their stents placed elsewhere, all stents placed for other indications other than stricture, and one case in which the removal date was not documented, the final cohort included 15 patients who had received a total of 48 stents for strictures after esophageal atresia repair.

### Demographics

The cohort included 3 girls and 12 boys. At the time of first stent intervention, the median age was 587 days and median weight was 9 kg. Of the 15 EA patients, 10 had a distal tracheoesophageal fistula, 6 had long-gap EA, and 2 had VACTERL association. See Table 1 for a summary of demographics.

**Table 1:** Baseline demographic and clinical characteristics, including original diagnosis and surgical history of all patients included in the study

Variable	n	%	Median	IQR
Total number of patients	15	100.0		
Male	12	80.0		
Female	3	20.0		
Age at first stent placement (months)			19.3	12.9–32.7
Weight at first stent placement (kg)			9.0	7.5–11.0
Original diagnosis				
Pure EA (Gross Type A)	2	13.3		
EA with distal TEF (Gross C)	10	66.7		
EA with proximal and distal TEF (Gross D)	2	13.3		
EA not classified	1	6.7		
Specific findings				
VACTERL association	2	13.3		
LGEA	6	40.0		
Prior surgical interventions (if reported)				
Foker	3	20.0		
Kimura	1	6.7		
Foker and Kimura	5	33.3		
Gastric pull-up	1	6.7		
Colon interposition	1	6.7		
Reanastomosis	1	6.7		

EA, esophageal atresia; VACTERL, acronym for a group of birth defects which tend to co-occur (vertebral, anorectal, cardiac, tracheoesophageal, renal, and limb anomalies); LGEA, long gap esophageal atresia; IQR, interquartile range

### Stent-related parameters

In the study, 48 stents were analyzed, a median of 3.2 per patient. Five patients received only one stent. Of the 48 stents, 22 (45.8%) were resorbable and 26 (54.2%) were non-resorbable. All resorbable stents were uncoated, and all non-resorbable ones were coated SEMS. Four patients received only SEMS, and three received only BDS. The others received a combination. Stent sizes were adapted to the patient where possible. Stent-related parameters, including sizes, are presented in Table 2.

**Table 2:** Stent characteristics, treatment modalities, and indications for removal

Variable	n	%	Median	IQR	Range
Total number of patients	15	100.0			
Total number of stents	48	100.0			
Number of BDS^a^	22	45.8			
Number of SEMS^b^	26	54.2			
Treatment with BDS only	3	20.0			
Treatment with SEMS only	4	26.7			
Mixed treatment BDS/SEMS	8	53.3			
Stent dimensions					
Total stent length (mm)			60		40–80
BDS length (mm)			60		40–80
SEMS length (mm)			60		40–80
Total stent body diameter (mm)			10		10–18
BDS body diameter (mm)			18		12–28
SEMS body diameter (mm)			10		10–14
Stent placement surgery time (min)			42,7^*^	± 13.5^**^	
Indication for SEMS removal	26	100.0			
Dislocation	8	30.8			
Newly developed fistula	1	3.8			
Dysphagia	2	7.7			
Exacerbated gastroesophageal reflux	1	3.8			
Non-stent related	1	3.8			
Regular removal	13	50.0			
Indication for BDS removal	1	100.0			
Dysphagia	1	100.0			
Total SEMS dwell time (days)			33	21–49	
Regular SEMS dwell time only (days)			35	22–51	
Time interval between stents (days)			118	69–160	

BDS, biodegradable stent; SEMS, self-expanding metal stent; IQR, interquartile range; SD, standard deviation

^a^SX-ELLA-degradable esophageal BD Stents (ELLA-CS, s.r.o., Hradec Králové, Czech Republic)

^b^BONASTENT® (Standard Sci-Tech Inc., Seoul, Korea) or aixstent® BDS (Leufen Medical, Berlin, Germany)

^*^Mean value reported for stent placement surgery time (min)

^**^Standard deviation reported for stent placement surgery time (min)

All placements were technically successful with no immediate complications. The mean procedure time was 42 min.

Of the 26 SEMS, removal due to complications occurred most often after dislocation (8 cases; 30.8%). Elective removal occurred in 13 cases (50%). SEMS remained in place for a median of 35 days. The median duration for electively removed SEMS was 33 days. Of the 22 BDS, only one was removed early due to severe dysphagia after 5 days. The remaining 21 (95%) stayed in place until dissolution. The median interval between consecutive stent placements was 118 days.

### Reinterventions and TTR

The results for reinterventions and Time to Reintervention (TTR) are presented in Table 3. Reintervention was required for 44 of the 48 stents (92%). For only 4 stents (8%), no reintervention was needed. In 28 cases (63.6%), dilation was the immediate reintervention (21 after SEMS, 24 after BDS). In 24 cases (32.9%), a new stent was inserted (12 after SEMS, 16 after BDS). On average, one dilatation was performed per stent. Topical medication (triamcinolone) was used in 10 cases.

**Table 3:** Overview of reinterventions, types of reintervention treatment, and Time To Reintervention (TTR)

Variable	n	%	Median	IQR
Reintervention required	44	91.7		
No Reintervention required	4	8.3		
Type of first reintervention				
Dilatation	28	63.6		
After SEMS	12			
After BDS	16			
Further stent	16	36.4		
After SEMS	10			
After BDS	6			
Further Dilatations per stent			1	0–3
Adjuvant treatments				
TA (triamcinolone)	10	22.7		
Time To Reintervention				
TTR total (days)			65	35–90
TTR–BDS (days)			77	47–123
TTR–SEMS (days)			49	27–69
	HR	CI		p^*^
TTR–BDS vs. TTR–SEMS	0.36	0.19–0.7		0.003

TTR, Time To Reintervention; BDS, biodegradable stent; SEMS, self-expanding metal stent; IQR, interquartile range; HR, hazard ratio; CI, 95% confidence interval

^*^A *p*-value < 0.05 was considered statistically significant

**Fig. 1:**
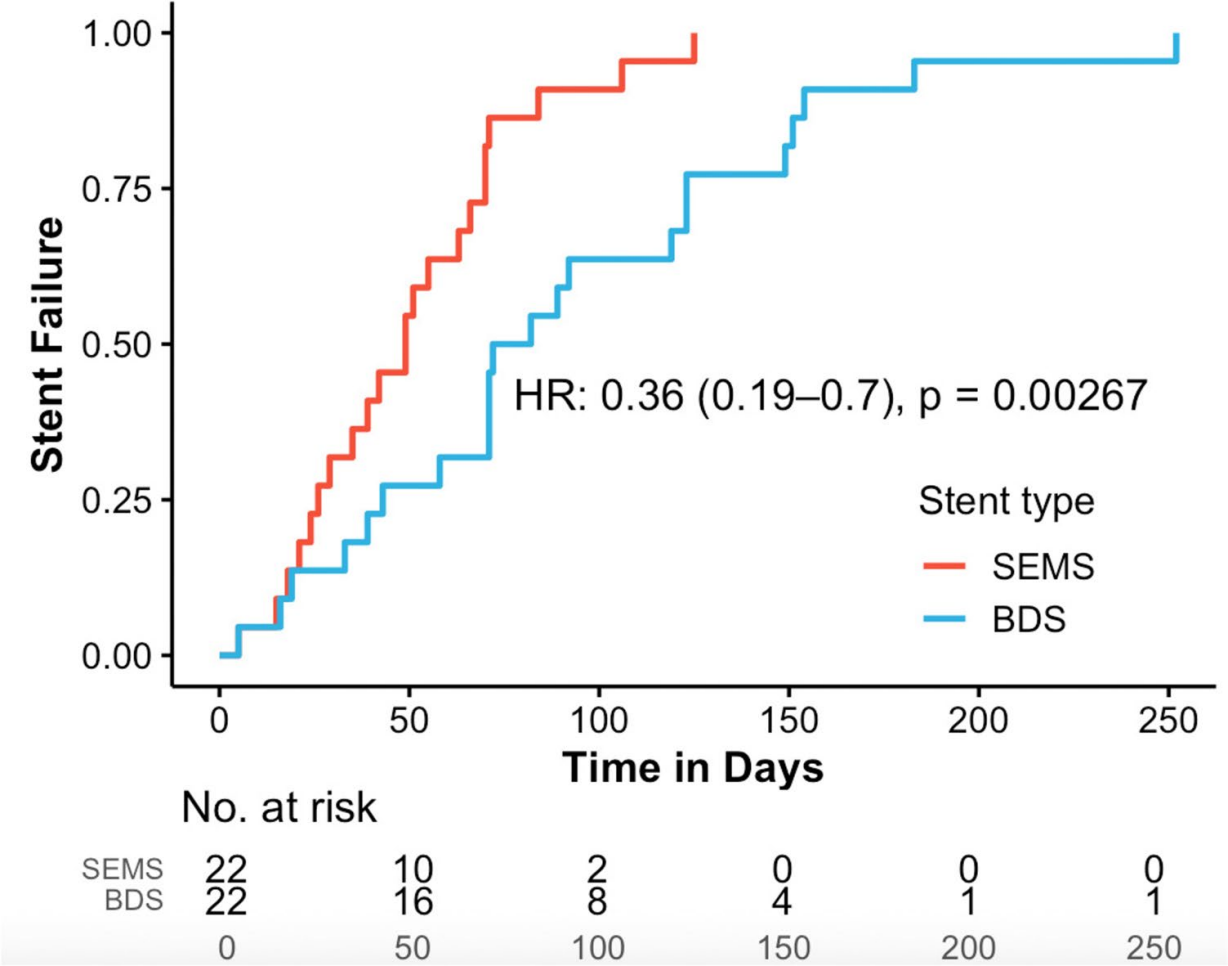
Kaplan–Meier plot showing stent failure over time after stent placement, stratified by stent type (self-expanding metal stents [SEMS, red] vs. biodegradable stents [BDS, blue]). Four stents that did not experience any failure were intentionally excluded from the Kaplan–Meier analysis and are therefore not represented as events in the curve; the number at risk table reflects the 44 stents included. Cox regression analysis demonstrated that patients treated with BDS had a 64% lower hazard of recurrence compared to those treated with SEMS (hazard ratio [HR]: 0.36; 95% confidence interval [CI]: 0.19–0.7; *p* < 0.00267)

The median TTR was 65 days overall (range: 15–260; IQR: 35–90). For BDS only, the median was 77 days (range: 15–252; IQR: 47–123), while for SEMS, it was 49 days (range: 19–260; IQR: 27–69). The Cox regression comparing BDS and SEMS with respect to recurrence and TTR showed that patients treated with BDS had a 64% lower hazard of recurrence (HR: 0.36; CI: 0.19–0.7; p < 0.003; see Fig. 1). The 4 stents without any observed reintervention during their entire follow-up period represent cases with sustained treatment success and were therefore not associated with an event. As no event occurred, these stents did not contribute to the estimation of median TTR or the Kaplan–Meier curve and were intentionally excluded from time-to-event analyses. As a result, the median, the survival curve and the number at risk table represent the 44 stents with at least one observed reintervention event. However, they are fully reported in Tables 4 and 5 to ensure transparent presentation of all treated stents and to allow appropriate reflection of their therapeutic success as well as their contribution to the observed complication profile.

**Table 4:** Therapeutic success per stent overall and for BDS/SEMS and Follow-up time

Therapeutic success^*^ per stent		
Variable	n	%
Procedural success overall	48	100.0
After 30 days	38	79.2
After 60 days	27	56.3
After 90 days	14	29.2
Procedural success BDS only	22	100.0
After 30 days	19	86.4
After 60 days	15	68.2
After 90 days	9	40.9
Procedural success SEMS only	26	100.0
After 30 days	19	73.1
After 60 days	12	46.2
After 90 days	5	19.2
	Median	Range
Follow-up time (months)	6	3–31

^*^Therapeutic success was defined as the absence of any further intervention combined with a patent esophageal lumen, confirmed by endoscopy, contrast study, or clinical assessment

**Table 5:** Overview of complications and their association with stent type

*Complications*						
	Total		SEMS		BDS	
Variable	n	%	n	%	n	%
Number of stents	48	100.0	26	100.0	22	100.0
No complications	28	58.3	16	61.5	12	54.5
Any Complications (multiple selection possible)	20	41.7	10	38.5	10	45.5
Dislocation	9	18.8	8	30.8	1	4.5
Obliteration	1	2.1	0	–	1	4.5
Erosion	1	2.1	0	–	1	4.5
Perforation	2	4.2	0	–	2	9.1
Bleeding	2	4.2	1	3.8	1	4.5
Newly developed fistula	1	2.1	0	–	1	4.5
Granulation tissue formation	10	20.8	3	11.5	7	31.8
Reflux	2	4.2	1	3.8	1	4.5
	*OR*		*CI*		p*	
Complication BDS vs. SEMS						
Complication any	1.33		0.36–4.91		0.770	
Dislocation	0.11		0.00–0.97		0.028	
Granulation tissue formation	3.48		0.99–24.2		0.052	

BDS, biodegradable stent; SEMS, self-expanding metal stent; OR, odds ratio; CI, = 95% confidence interval

^*^A *p*-value < 0.05 was considered statistically significant

### Therapeutic success and complications

Procedural success was 79.2% at 30 days, 56.3% at 60 days, and 29.2% at 90 days. Table 4 summarizes the results. The median follow-up was 6 months. In 4 patients, no further procedure was performed. Endoscopic follow-up treatment was performed in 11 patients, and no patients required surgical revision of their esophageal stenosis.

Complications occurred with 20 stents (41.7%). Table 5 provides an overview. The most common was dislocation (9 cases, 18.8%), with 8 being SEMS and 1 BDS. The second most common was granulation tissue formation (10 cases, 20.8%). Other complications included stent obliteration (1 case), erosion (1 case), perforation (2 cases), and bleeding (2 cases), and a new TEF (1 case). Severe complications included an unrelated death in the SEMS group and a cerebral abscess in the BDS group.

Statistical analysis showed no association between stent type and overall complications, obliteration, erosion, perforation, bleeding, fistula, or reflux. However, a significant association was found between dislocation and SEMS use (OR: 0.11; CI: 0.00–0.97, *p* < 0.028), and a trend toward more granulation tissue formation with BDS use (OR: 3.48; CI: 0.99–24.2; *p* = 0.052).

## Discussion

To our knowledge, this is one of the largest studies investigating the use of stents in the treatment of benign esophageal diseases in pediatric patients, and the first study directly comparing BDS and SEMS devices.

### Success of stenting therapy in general

Previous studies show considerable variation. Tandon et al. [9] found 8% of patients needed no further treatment, 44% needed dilations, and 32% required surgical revision. Our cohort showed a slightly better outcome: 20% successfully treated with stents alone, 55% needing further endoscopic intervention, and 25% undergoing surgery.

The largest study to date, by Baghdadi et al. [14], focused on stenosis and excluded BDS. Their success criterion—avoiding surgical resection within 12 months—showed stenting prevented surgery in 41% of cases. Applying this to our cohort, 80% avoided surgery.

Our findings, in line with Baghdadi et al. [14], suggest stents significantly reduce the need for surgery. However, recurrent stenosis remains common, indicating stents primarily extend intervention-free periods rather than providing a definitive solution.

### BDS in a pediatric cohort

Data on BDS use in children is scarce. This study found that BDS achieved a longer median TTR compared to SEMS (71 vs. 35 days). This has clinical implications: BDS may minimize the burden of repeated anesthesia and endoscopic interventions [15, 16], potentially improving quality of life and reducing healthcare resource utilization [17].

However, direct comparison is limited. Indications differed: BDS were used only for strictures, while SEMS were also used for more complex lesions. SEMS often had to be removed electively, which may have artificially shortened their TTR. More SEMS (*n* = 26) were removed prematurely due to complications, suggesting better size adaptation could have extended TTR. Many patients received both stent types, further complicating comparisons.

BDS still appear to provide a longer intervention-free period. Kailla et al. [18] reported a TTR of 100–260 days in adults. For BDS-only stenosis treatment, our success rate was 50%. A similar study reported 66.6% [4]. These findings suggest resorbable stents may be a promising alternative to invasive surgical resection. Further prospective studies are warranted.

### Comparison with alternative therapies

Stents aim to avoid invasive surgical revision [19]. At an average of 42 min, stent placement is a less invasive procedure with less anesthesia time. All placements were technically successful and free of immediate complications. Given a median of 2.5 stents per patient and a long-term success rate without surgery of 30%, attempting stent treatment seems justified. Some advocate for surgical revision after 7–10 endoscopic interventions [20, 21]. However, surgical repair does not guarantee success: Koivusalo et al. [22] reported that 10% of patients did not survive revision surgery, and 12% needed more reoperations. Surgical repair is also complicated by mediastinal scarring [23, 24]. We agree that a non-operative endoscopic approach should be preferred before surgical repair [25].

Balloon dilatation or bougienage is usually the primary approach to postoperative anastomotic strictures. In a previous nationwide registry study, we found that there were a total of 188 endoluminal procedures per 100 esophageal atresia anastomoses during the first year of life, the vast majority of them simple dilatations [26]. Al-Sarkhy et al. [27] found balloon dilation required a median of 3 sessions for resolution, with a success rate of 70–100%, and a perforation rate around 1–2%. In our center, we generally perform around 10 dilatations before we consider stent placement. However, the data in this study shows that in complex cases, early stent placement may offer longer intervention-free periods than repeated dilation alone. Conversely, BDS with a TTR of 71 days could offer a longer intervention-free period. In Al-Sarkhy et al.'s study, 64% of patients eventually needed surgical revision. Other studies noted that patients with long-gap atresia still required surgical resection after numerous dilations (up to 15) [28]. Predictors for failure of dilation therapy in recurrent strictures include long-gap atresia, stricture length ≥ 10 mm, anastomotic leakage, and ≥ 7 balloon dilations [29].

### Complications

Our study found stent use is generally safe. While 48.1% of stents had complications, they were usually minor. Severe complications like bleeding or perforation were rare (3.8% each). This aligns with a systematic review reporting few severe stent-related complications [30]. A study on SEMS and SEPS reported a significantly higher rate of 71%, including two deaths [30], which the authors attributed to inexperience and complicated cases. With sufficient expertise, stents can be used safely [14].

Dislocation was the most common complication, with SEMS showing a significantly higher rate. Tandon et al. [9] reported a lower rate of 14.4%. The use of suboptimal-sized, adult stents may have contributed to our higher rate. Fully coated stents have a higher dislocation risk as the coating prevents tissue ingrowth [31, 32]. However, uncoated, non-resorbable stents are not recommended in children due to ingrowth. Our BDS dislocation rate was exceedingly low at 4.5%, consistent with adult studies [18]. Others have also suggested BDS may be preferable due to lower dislocation rates [33].

Another significant finding was tissue hyperplasia, which was significantly more common with BDS. This can lead to stenosis and the need for intervention. Karakan et al. [34] identified this as a main limiting factor for BDS. Granulation tissue was more frequent in our caustic injury patients. We hypothesize that underlying tissue damage and mechanical irritation played a role.

The choice of stent type involves balancing typical complications. A direct comparison of dislocation and tissue hyperplasia concludes that dislocation inevitably requires intervention, while hyperplasia only rarely leads to clinically apparent restenosis. Therefore, BDS should be the primary choice when available in the appropriate size, particularly for isolated stenosis.

### Dwell time

For non-resorbable stents, the optimal dwell time is a central issue. The European Society of Gastrointestinal Endoscopy ESGE recommends 6–8 weeks, with a maximum of 3 months for SEMS, noting the weak evidence [5]. Manufacturers suggest slightly shorter durations of 4–6 weeks. The key is balancing the risk of stent-induced complications with the time needed for healing [35]. Prolonged dwell times increase the risk of tissue ingrowth and perforation. For BDS, the dissolution time is a key advantage. They can remain in place until they dissolve, providing a longer period of treatment without the need for a separate removal procedure and the associated risks.

### Economic aspect

In our environment, SEMS currently cost around 700 to 900 Euros, while BDS devices cost about twice as much, around 1400 to 1900 Euros (purchasing data for the stents from the administrative office of our university hospital). The use of custom-made stents results in even higher costs. Three-dimensional printing may become an option once resorbable materials can be printed at a reasonable price. Until then, however, availability remains inconsistent, and the use of poorly sized prefabricated stents is an issue when using products made for adults in an "off-label" manner. However, the higher costs may be offset by savings from reduced hospitalization rates, lower anesthesia and operating room costs, faster patient recovery, and less parental absenteeism from work [4].

### Limitations

This study has several limitations. It is a retrospective analysis from a small, single-center cohort, which limits generalizability. While it represents a large pediatric cohort, the sample size is still small. The lack of randomization means treatment choices were influenced by factors like stent availability and surgeon preference. The off-label use of stents and varied sizes also complicates direct comparisons. Furthermore, the definition of therapeutic success is complex in these patients, and the decision for surgical revision is highly subjective. Finally, the long-term outcomes beyond the recorded follow-up intervals and patient-reported quality of life were not systematically assessed.

Although the observed TTR difference reached statistical significance in the Cox regression analysis, the relatively small sample size limits the statistical power of the analysis, and the results should therefore be interpreted with caution. Further studies with larger cohorts are warranted to confirm these findings. Additionally, four stents did not require any reintervention during the entire follow-up period and were therefore not associated with an observable event. As these cases did not allow estimation of time-to-reintervention, they were not included in the Kaplan–Meier or median TTR analyses. While this approach ensured comparability of event-based outcomes, it may have led to a conservative estimation of intervention-free survival, particularly in the SEMS group.

Therefore, future prospective, randomized, and multicenter studies are needed to confirm our findings and provide more definitive guidance.

## Conclusion

In this retrospective study of children with benign esophageal diseases, we found that stent treatment is an effective and safe approach that significantly reduces the need for surgical revision. BDS are associated with a longer TTR compared to SEMS and have a lower risk of dislocation. While BDS show a higher rate of granulation tissue formation, this complication is often self-limiting and less clinically significant than dislocation. These findings suggest that BDS may be the preferred stent for isolated strictures, provided they are available in the appropriate size. The use of stents in general, even when multiple interventions are needed, is a valuable alternative to surgical reconstruction, particularly in complex cases.

## Data Availability

All rawdata pertaining to this study are available from the senior author (OJM) upon reasonable request.
